# Geospatial epidemiology of Staphylococcus aureus in a tropical setting: an enabling digital surveillance platform

**DOI:** 10.1038/s41598-020-69312-4

**Published:** 2020-08-05

**Authors:** T. M. Wozniak, W. Cuningham, S. Buchanan, S. Coulter, R. W. Baird, G. R. Nimmo, C. C. Blyth, S. Y. C. Tong, B. J. Currie, A. P. Ralph

**Affiliations:** 1 Menzies School of Health Research, Global & Tropical Health, Charles Darwin University, Darwin, Northern Territory Australia; 2 Queensland Health, Communicable Diseases Branch, Brisbane, Queensland Australia; 3 Territory Pathology, Northern Territory Government, Darwin, Northern Territory Australia; 4 Pathology Queensland Central Laboratory, Griffith University School of Medicine, Brisbane, Queensland Australia; 5 Wesfarmers Centre of Vaccines and Infectious Diseases, Telethon Kids Institute, University of Western Australia, Perth, Western Australia Australia; 6 Department of Infectious Diseases, Perth Children’s Hospital, Perth, Western Australia Australia; 7 PathWest Laboratory Medicine, Perth, Western Australia Australia; 8 Victorian Infectious Disease Service, The Royal Melbourne Hospital and Doherty Department University of Melbourne, at the Peter Doherty Institute for Infection and Immunity, Melbourne, Victoria Australia; 9 Department of Infectious Diseases, Royal Darwin Hospital, Darwin, Northern Territory Australia

**Keywords:** Diseases, Health care, Pathogenesis

## Abstract

Delivery of information to clinicians on evolving antimicrobial susceptibility needs to be accurate for the local needs, up-to-date and readily available at point of care. In northern Australia, bacterial infection rates are high but resistance to first- and second-line antibiotics is poorly described and currently-available datasets exclude primary healthcare data. We aimed to develop an online geospatial and interactive platform for aggregating, analysing and disseminating data on regional bacterial pathogen susceptibility. We report the epidemiology of *Staphylococcus aureus* as an example of the power of digital platforms to tackle the growing spread of antimicrobial resistance in a high-burden, geographically-sparse region and beyond. We developed an online geospatial platform called HOTspots that visualises antimicrobial susceptibility patterns and temporal trends. Data on clinically-important bacteria and their antibiotic susceptibility profiles were sought from retrospectively identified clinical specimens submitted to three participating pathology providers (96 unique tertiary and primary healthcare centres, n = 1,006,238 tests) between January 2008 and December 2017. Here we present data on *S. aureus* only. Data were available on specimen type, date and location of collection. Regions from the Australian Bureau of Statistics were used to provide spatial localisation. The online platform provides an engaging visual representation of spatial heterogeneity, demonstrating striking geographical variation in *S. aureus* susceptibility across northern Australia. Methicillin resistance rates vary from 46% in the west to 26% in the east. Plots generated by the platform show temporal trends in proportions of *S. aureus* resistant to methicillin and other antimicrobials across the three jurisdictions of northern Australia. A quarter of all, and up to 35% of methicillin-resistant *S. aureus* (MRSA) blood isolates in parts of the northern Australia were resistant to inducible-clindamycin. Clindamycin resistance rates in MRSA are worryingly high in regions of northern Australia and are a local impediment to empirical use of this agent for community MRSA. Visualising routinely collected laboratory data with digital platforms, allows clinicians, public health physicians and guideline developers to monitor and respond to antimicrobial resistance in a timely manner. Deployment of this platform into clinical practice supports national and global efforts to innovate traditional disease surveillance systems with the use of digital technology and to provide practical solutions to reducing the threat of antimicrobial resistance.

## Introduction

Antimicrobial resistance (AMR) imposes a substantial burden including poorer clinical outcomes and higher healthcare costs for patients infected with resistant compared to susceptible organisms^1–3^. Quantifying the magnitude of AMR over time and space is essential to better target public health priorities. Despite the high ssoverall health status of many Australians, rural and regional areas within this country continue to experience comparatively poor health outcomes^4^. Northern Australia comprises half the Australian landmass but includes only 5% (1.3 million) of the total Australian population. This region has a tropical climate and is home to 30% of Australia’s Aboriginal and Torres Strait Islander people^4^. It spans three separate jurisdictions, each with its own health program and multiple electronic health record systems, limiting cross-jurisdictional information flow. Despite the sparse population it has some of the highest burden of infectious diseases including methicillin-resistant *Staphylococcus aureus* (MRSA)^5–7^.

Current efforts to track and respond to AMR systematically in northern Australia and other regional settings are insufficient^8–10^. In addition, many regional and remote hospitals also do not have the specialist services that support antimicrobial stewardship^11^. Combined, this leaves a significant gap in health services’ ability to initiate appropriate, timely responses to changing AMR epidemiology. To achieve enhanced AMR surveillance in Northern Australia, cross-jurisdictional collaboration with data sharing is required and the use of innovative geospatial information systems, can support these efforts.

We developed an online AMR surveillance platform that integrates existing but fragmented susceptibility data, for visualization and analysis across time and space. Northern Australia was selected due to the paucity of existing data from this region^12^ especially from primary care settings, the unique endemic pathogens, and concerns about high rates of antibiotic resistance in *S. aureus* in this region. We describe, using geospatial epidemiology of *S. aureus* as an example, a digital platform used as a disease surveillance tool. The intent of the HOTspots surveillance platform is to be a dynamic and collaborative platform used by healthcare practitioners, policy makers and therapeutic guideline developers.

## Methods

### Setting

**Figure 1:**
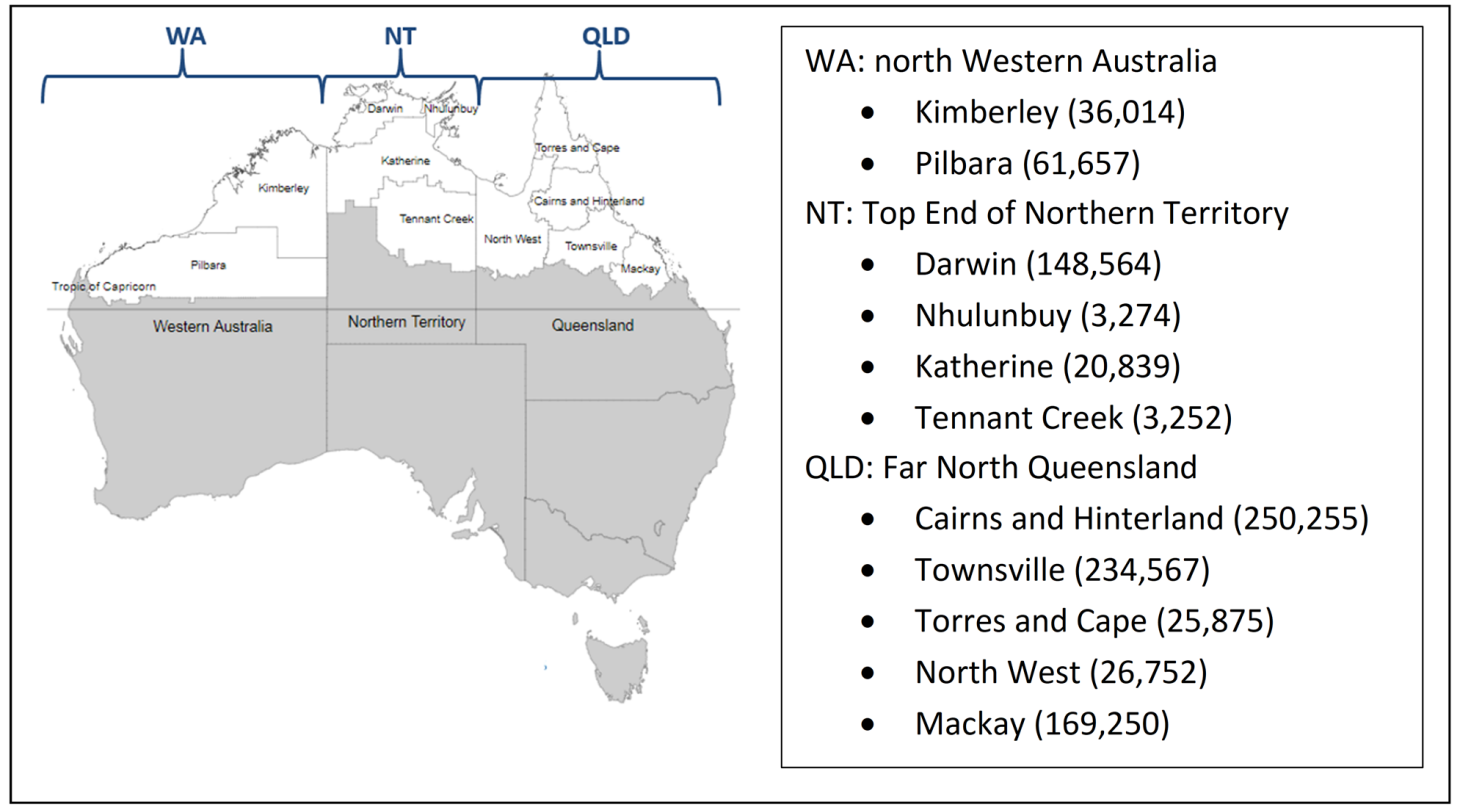
Selected geographical regions and population density in 2017. Map generated by HOTspots platform https://amrhotspots.com.au/.

The four major laboratories serving primary and tertiary institutions in northern tropical Australia, defined as areas north of latitude 23 degrees, were invited to participate. Northern Australia includes three separate jurisdictions: Western Australia, Northern Territory and Queensland. The Australian Bureau of Statistics (ABS) boundaries were categorised as Statistical Area Level 3, with populations of 30,000 to 130,000 people^13^. Participating regions comprised two northern regions of north Western Australia (hereafter referred to as WA), four regions of the Top End of Northern Territory (hereafter referred to as NT) and five regions of far north Queensland (hereafter referred to as QLD) (Fig. 1).

### HOTspots surveillance platform

The HOTspots platform (https://amrhotspots.com.au/) is based on geo-coordinates providing spatial localization of resistance information. It is a custom-built platform with Hypertext Preprocessor (PHP), Hypertext Markup Language (HTML), and JavaScript with D3.js visualisation library for the frontend and MySQL (a programming language) for the backend. It is delivered on a Linux Server and is accessible on any world wide web search engine. Microbiological data provided from the participating laboratories include: year of test, location of sample collection, sample type (blood, urine or swab), organism isolated and susceptibility to a list of pre-specified antibiotics (Table 1). These data are entered as a line listing of individual de-duplicated isolates.

**Table 1:** List of organisms and antibiotics included in the HOTspots tool.

Organism	Antibiotic class	Antibiotic
*Escherichia coli* and *Klebsiella pneumoniae*	Beta-lactam	Amoxicillin and enzyme inhibitor Cefazolin Ceftazidime Ceftriaxone
	Quinolone	Ciprofloxacin
	Carbapenem	Meropenem
	Aminoglycoside	Gentamicin Amikacin Tobramycin
*Pseudomonas aeruginosa*	Beta-lactam	Ceftazidime
	Quinolone	Ciprofloxacin
	Aminoglycoside	Gentamicin Amikacin Tobramycin
	Carbapenem	Meropenem
*S. aureus*	Beta-lactam	Methicillin
	Macrolide	Erythromycin
	Lincosamide	Clindamycin
	Folate inhibitor	Sulfamethoxazole-trimethoprim (SXT)

A multifunctional search toolbar allows quick and direct searching of the year, organism and antibiotic of interest for visualization. Data can be visualized on a map, or as a plot of percent resistance to a given antibiotic over time. The map displays raw proportions for a given year whilst the line plot displays temporal trends using a 3-year moving average. The region of interest can be expanded or contracted. For years with few isolates collected and tested (< 15 tests), these data within the region of interest are aggregated or excluded if all years have < 15 tests. Deployment of HOTspots into clinical practice in 2020 will permit 6-monthly data updates entered automatically into the platform.

### Microbiological data

Organisms and antibiotics were selected for inclusion in HOTspots through discussion with the study team and local experts to represent those of greatest clinical relevance (Table 1). Participating laboratories provided data on all clinical specimens where susceptibility testing was performed during the study period. Since clinical information or additional specimen information (e.g. swab site; Gram stain and microscopy) was unavailable, the classification as community or nosocomial-onset and clinical significance of bacterial isolates could not be determined. Information on sample type (i.e. blood, swab and urine) was available for all organisms in NT and QLD and in WA for *S. aureus* isolates only. Results were restricted to the first bacterial isolate per patient per year and missing or erroneous entries were excluded. All resistant and intermediate results were combined as ‘Resistant’ for purposes of resistance phenotype analysis.

Participating laboratories are accredited under regularly audited national testing guidelines (National Association of Testing Authorities); and are members of the National Quality Assurance and Quality Control program run by the Royal College of Pathologists of Australasia External Quality Control Assurance program. These programs have been in place for over 30 years and ensure a high concordance with reproducibility of microbiology susceptibility results between different laboratories.

Two widely used international susceptibility method systems, Clinical and Laboratory Standards Institute (CLSI) and European Committee on Antimicrobial Susceptibility Testing (EUCAST), were used by the participating laboratories. Susceptibility results were determined by VITEK 2 (bioMerieux, France). Data from WA were provided with CLSI-interpreted values (Susceptible, Intermediate and Resistant). Data from QLD was provided with CLSI-interpreted values (Susceptible, Intermediate and Resistant) for years 2008 to 2012, and EUCAST interpreted values for years 2012 onwards. For isolates from NT, we applied the 2017 CLSI M100-S27 Performance Standards for Antimicrobial Susceptibility Testing (27th Edition) to the provided minimum inhibitory concentration values for each organism. In all laboratories, clindamycin resistance was inferred from erythromycin resistance and a D-test only performed upon request (referred to as inducible-clindamycin). Methicillin resistance in *S. aureus* was inferred from resistance to oxacillin in laboratories in the WA, cefoxitin in NT laboratories and flucloxacillin and cefoxitin in QLD.

### Analysis

We used Stata 15.1 for data management and descriptive statistics. Proportion resistant was calculated as the number of samples resistant to the antibiotic divided by number of susceptibility tests. The validity of a subset of HOTspots susceptibility data was assessed by comparing MRSA reported in the Top End Health Service (TEHS, Northern Territory) antibiogram as a reference^14^. The TEHS antibiogram included all isolates tested during November 2016 to April 2017. An agreement statistic^15^ was calculated for MRSA where M denotes percent agreement, R denotes the reference susceptibility and A denotes the HOTspot susceptibility.

$${\text{M}} = {1 }{-} \, [({\text{sum}}\left| {{\text{R }}{-}{\text{ A}}} \right|) \, /{\text{sumR}})].$$

### Ethics

The study was conducted and approved by provided by the Human Research Ethics Committee of the Northern Territory Department of Health and Menzies School of Health Research (HREC-2018-3084) as well as the Queensland Health Public Health Act 2005 (Section 280). All data were analysed in strict compliance with the requirements of the National Statement on Ethical Conduct in Human Research (2007) guidelines.

## Results

The contributing pathology providers collectively have 96 specimen collection sites (hospitals and primary healthcare centers) and provided 1 006 238 susceptibility tests. Resulting data were derived from primary and tertiary care for the WA and QLD and tertiary care only for the NT. Characteristics of the databases which form the basis of the HOTspots platform are described in Table 2.

**Table 2:** Database characteristics for HOTspots surveillance tool.

	WA	NT	QLD
Years of analysis	2014–2017	2012–2017	2008–2017
Number of tests	94,919	173,909	737,410
Unique locations	6	4	86
Blood	Not specified except for *S. aureus*	9,868	31,998
Urine		81,527	255,886
Swab		80,776	392,753
Laboratory standards	CLSI	CLSI	CLSI (2008–2012) EUCAST (2012–current)

WA, north Western Australia; NT, top end of Northern Territory; QLD, far north Queensland; CLSI, Clinical and Laboratory Standards Institute; EUCAST, European Committee on Antimicrobial Susceptibility Testing.

### HOTSpots platform development and validation

**Figure 2:**
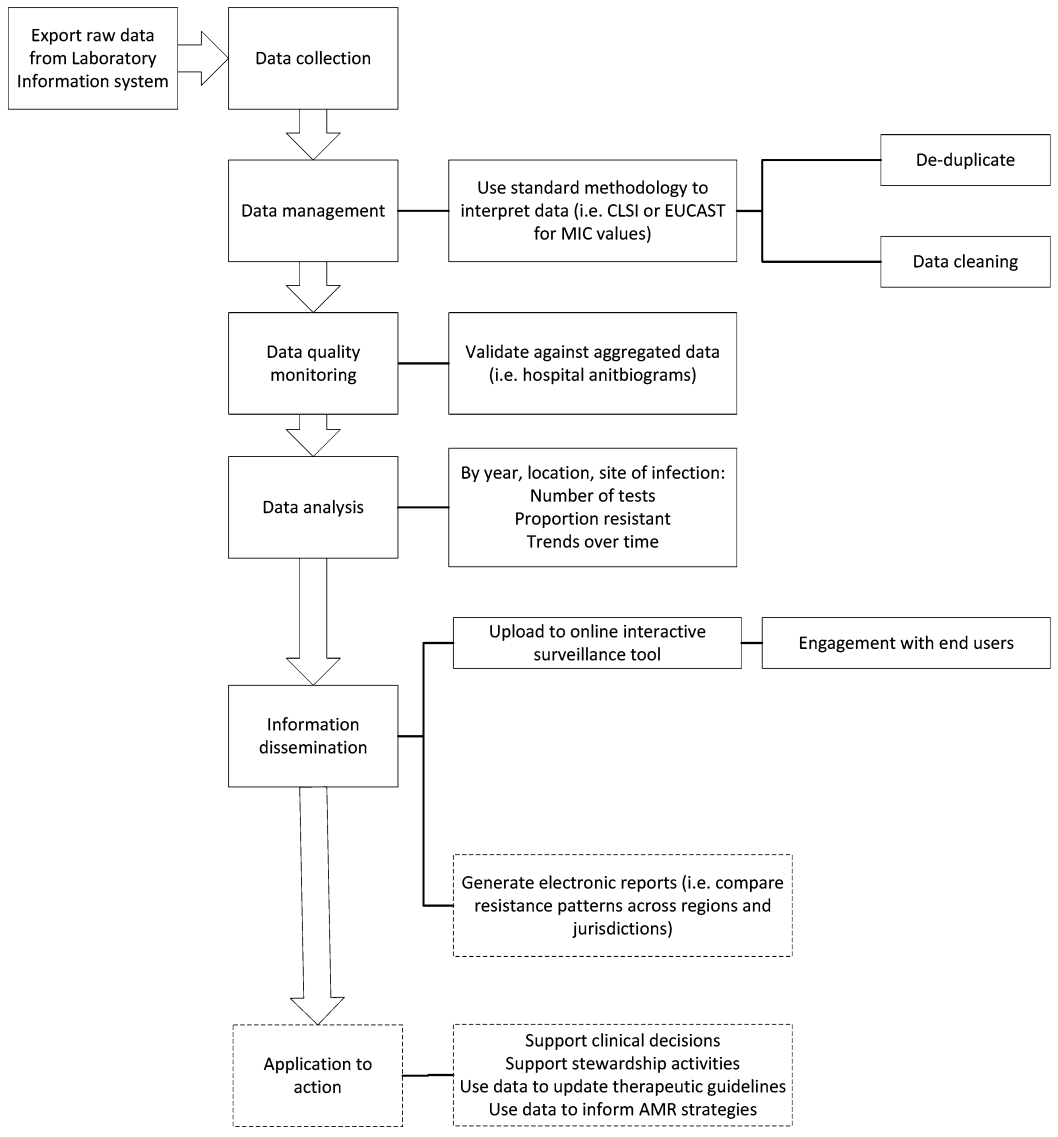
Data flow and processes of the HOTspots surveillance tool.

Surveillance data sent to the HOTspots surveillance team were coordinated and cleaned. Deidentified data are sent in a format which is directly extracted from the Laboratory Information System from each of the pathology providers. These data are managed through a pipeline described in Fig. 2. To cross-check a subset of data, agreement between the proportion of MRSA bloodstream isolates in NT was compared with the 2017 hospital antibiogram. This was calculated as 94·9% (1 − [(34·6 − 32·85)/34·6)] and interpreted as showing strong agreement.

### Epidemiology of S. aureus and methicillin-resistance by geographic region

**Figure 3:**
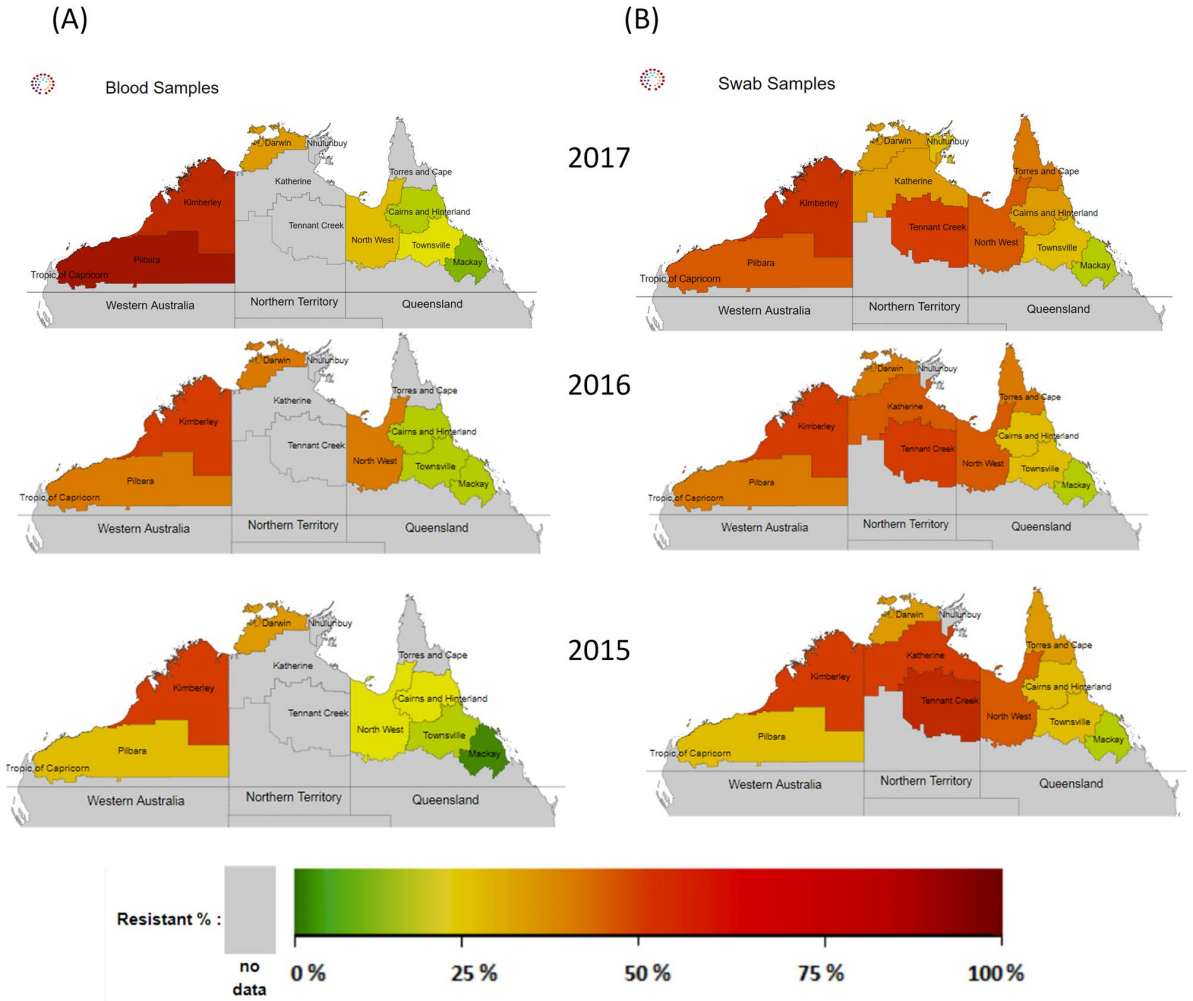
HOTspots output for resistance among *Staphylococcus aureus* isolates to methicillin in blood cultures (**A**) and swab samples (**B**), northern Australia, 2015–2017. Maps generated by HOTspots platform https://amrhotspots.com.au/.

During the study period, we obtained 137 723 *S. aureus* isolates for analysis, 90% of these were from swabs (n = 125 740) and the remainder were blood (n = 3 462), urine (n = 2 696) and unspecified (n = 3 823) (Table 3). Methicillin resistance was identified in 35% (n = 48 203) of *S. aureus* isolates overall and was highest in blood cultures (37%) compared to other isolates (Table 3). There was variation between (Table 3) and within jurisdictions (Table 3, Fig. 3) in the patterns of MRSA. Between jurisdictions, MRSA was highest in WA (46.8%), compared to NT (34%) and QLD (26%) (Table 3). Within jurisdictions, WA data demonstrates an increase in MRSA in the Pilbara region blood (25% in 2015 to 75% in 2017, Fig. 3A) and swab isolates (29% in 2015 to 41% in 2017, Table 3). With the available data we could not determine NT intra-jurisdictional patterns of resistance in blood cultures of *S. aureus* (Fig. 3A). However, data from swab isolates suggests a decline in methicillin resistance in both Darwin (35% in 2015 to 24% in 2017) and Tennant Creek (55% in 2015 to 34% in 2017) regions of NT (Table 4, Fig. 3B). In QLD, proportion of swab isolates resistant to methicillin has remained stable since 2015, except for ‘Torres and Cape’ and ‘Cairns and Hinterland’ regions, where MRSA has increased by 5% (Table 4).

**Table 3:** *Staphylococcus aureus* isolates with available susceptibility data and proportion resistant to methicillin, 2008–2017.

	WA (2014–2017)	NT (2012–2017)	QLD (2008–2017)	All regions
***S. aureus, N (% MRSA)***				
All sites	21,223(46%)	17,685 (34%)	98,815 (26%)	137,723 (35%)
Blood	144 (54%)	575 (36%)	2,743 (21%)	3,462 (37%)
Swab	21,079 (46%)	16,763 (34%)	87,898 (26%)	125,740 (35%)
Urine	··	347 (33%)	2,349 (16%)	2,696 (18%)
Unspecified	··	··	3,823 (23%)	3,823 (23%)

WA, north Western Australia; NT, top end of Northern Territory; QLD, far north Queensland; N: number tested.

**Table 4:** Susceptibility patterns of MRSA swab isolates in northern Australia by region,* 2015–2017.

Number tested (% MRSA)		2015	2016	2017
WA	Kimberley	3,871 (50%)	3,698 (52%)	3,826 (53%)
	Pilbara	1,373 (29%)	1,513 (39%)	1,412 (41%)
NT	Darwin	2,276 (35%)	2,649 (37%)	1,899 (24%)
	Katherine	179 (45%)	85 (42%)	16 (44%)
	Tennant Creek	198 (55%)	230 (49%)	175 (34%)
QLD	Torres and Cape	2,206 (33%)	2,929 (37%)	2,947 (38%)
	Cairns & Hinterland	2,554 (26%)	2,847 (28%)	2,906 (31%)
	Townsville	2,316 (27%)	2,412 (27%)	2,652 (27%)
	North West	1,125 (44%)	1,331 (45%)	1,196 (44%)
	Mackay	1,057 (17%)	1,072 (16%)	1,200 (16%)

WA, north Western Australia; NT, Top End of Northern Territory; QLD, far north Queensland.

*Data were not available for Nhulunbuy region in NT.

### Second-line S. aureus antibiotic resistance

**Figure 4:**
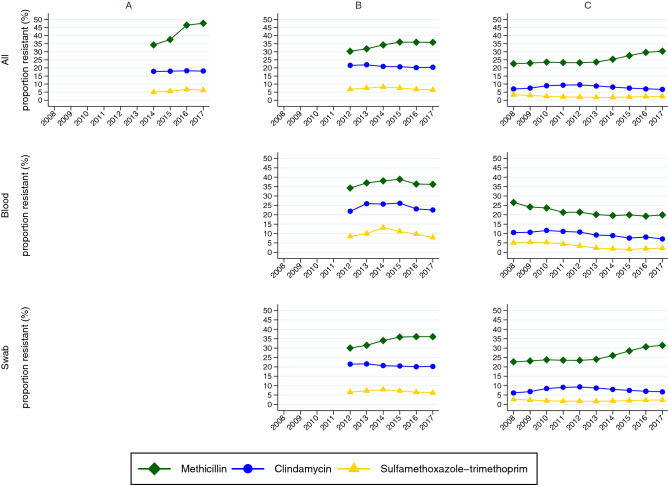
Proportion of *S. aureus* isolates resistant to given antibiotics in the WA (**A**), NT (**B**) and QLD (**C**). Top panel: all samples; middle panel: blood cultures and lower panel: swab samples, 2008–2017.

*S. aureus* resistance to inducible-clindamycin and sulfamethoxazole-trimethoprim (SXT) was higher in WA and NT and lower in QLD (Fig. 4, Table 5). Resistance to these second-line agents was more common in *S. aureus* isolates that were methicillin resistant than methicillin sensitive (Table 5). Resistance to inducible-clindamycin among MRSA isolates was 10% in QLD compared to 26% in NT and 20% in WA. MRSA resistant to SXT was the same in QLD and NT (5% of all isolates) and almost three times higher in WA (14%, n = 1 345 resistant isolates). There were 27 MRSA blood cultures tested for SXT between 2014 and 2017 in WA, and none were found to be resistant to SXT.

**Table 5:** Second-line antibiotic resistance rates according to methicillin susceptibility in *Staphylococcus aureus* isolates, northern Australia, 2008–2017.

		Clindamycin, number tested (% resistant)			SXT, number tested (% resistant)		
		All isolates	Swab	Blood	All isolates	Swab	Blood
WA	All isolates	20,871 (18%)	20,871 (18%)	··	20,922 (7%)	20,873 (7%)	49 (0%)
	MSSA	11,299 (19%)	11,299 (19%)	··	11,311 (1%)	11,289(1%)	22 (0%)
	MRSA	9,572 (20%)	9,572 (20%)	··	9,611 (14%)	9,584 (14%)	27 (0%)
NT	All isolates	17,680 (21%)	16,758 (21%)	575 (25%)	17,683 (7%)	16,761 (2%)	575 (9%)
	MSSA	11,628 (19%)	11,032 (19%)	365 (19%)	11,629 (1%)	11,033 (2%)	365 (1%)
	MRSA	6,052 (26%)	5,726 (25%)	210 (35%)	6052 (5%)	5,726 (16%)	210 (25%)
QLD	All isolates	96,967 (8%)	86,629 (8%)	2,743 (9%)	90,960 (2%)	80,820 (2%)	2,728 (3%)
	MSSA	71,798 (7%)	63,720 (7%)	2,160 (7%)	65,918 (1%)	58,007 (1%)	2,146 (1%)
	MRSA	25,169 (10%)	22,909 (9%)	583 (19%)	25,042 (5%)	22,813 (4%)	582 (11%)

WA, north Western Australia; NT, Top End of Northern Territory; QLD, far north Queensland; SXT, sulfamethoxazole-trimethoprim; MSSA, methicillin-sensitive *S. aureus*; MRSA, methicillin-resistant *S. aureus.*

### Longitudinal trends in S. aureus antimicrobial resistance

The proportion of *S. aureus* resistant to methicillin increased overall during the study period. However in blood, MRSA was stable or decreasing over time for QLD and NT where the data are available (Figs. 3, 4). The increase in MRSA in QLD swab samples likely originates from either the ‘Torres and Cape’ or ‘Cairns and Hinterland’ region (Fig. 3B, Table 4). The proportion of all *S. aureus* resistant to other major antibiotics was stable or decreased (Fig. 4).

## Discussion

We demonstrate the feasibility and utility of a digital geospatial surveillance platform for displaying antimicrobial resistance data. This prototype forms the basis of a platform that will be able to host increasingly comprehensive microbiological data. It offers a mechanism to support clinical and public health decision-making and guideline development. The urgent need to improve antimicrobial stewardship in the remote and disadvantaged regions of central and northern Australia has been recently highlighted^8^.

A comprehensive epidemiology of *S. aureus* across tropical north of Australia has not previously been reported. These findings show striking geographical variation in *S. aureus* susceptibility across northern Australia with the proportion of MRSA ranging from 46% in WA to 26% in QLD and an overall 35% across this tropical region. This is a two-fold higher proportion of MRSA than has been reported from southern parts of Australia (range from 9.5 to 20.5%)^16^. To fulfil Australia’s first National AMR Strategy and implementation plan^17^, a high-quality evidence-base is needed to support geospatially-representative data collection, coordinated surveillance activities and targeted policy decisions. Repeated calls for a new Australian national coordinating centre on AMR have to date gone unheeded^18^.

Australia has already invested heavily in understanding the epidemiology of AMR, with some key national initiatives^12^. From these combined efforts, it is evident that AMR is increasing across Australia, however available data tells us little about interjurisdictional variation and the complexity of AMR in community settings. *S. aureus* provides an example of an organism with patterns of resistance that are geographically heterogenous, yet publications have only focused on specific regions in individual studies^5,6,19–21^. These studies report rates of community-associated MRSA incidence ranging from 16 per 100,000 population to 81 per 100,000 population, depending on the study population and region. This does not reflect the heterogeneity that can occur between urban and remote settings and the distribution of factors that likely determine transmission of infectious diseases. The distinction is important because it helps determine the most appropriate level for clinical and policy intervention. Spatial epidemiology can fill the gaps in traditional surveillance and equip healthcare professional with the data analytics to effectively make decisions and policy recommendations to improve patient and population-level outcomes^22^.

The temporal trends in *S. aureus* epidemiology seen across northern Australia relate to changes in dominant MRSA clones both within and between jurisdictions^16^. The most common community-associated MRSA clone in Australia is now ST93-MRSA, which most likely arose from northern Australia in the late 1980s^23^ and spread east. In 2017, this strain accounted for 33% community-associated MRSA ranging from 0% in Tasmania to 74% in the Northern Territory^16^. Community-associated MRSA clones have emerged serially from strains of methicillin-sensitive *S. aureus* (MSSA)^6,23^ and over the years have diversified and increased in frequency across the globe^23^.

Our data highlight the problem of MRSA resistant to inducible-clindamycin and SXT, commonly relied on as oral second-line options for community MRSA skin and soft tissue infections, or as first-line agents for people with MSSA infection with intolerance to beta-lactams. SXT was introduced in 2014 as an alternative agent to intramuscular penicillin for the treatment of streptococcal skin infections, highly endemic in this setting^24^. Concerns about a subsequent rise in SXT resistance in *S. aureus* have been expressed^25^, but we did not find this in our analysis. The SXT resistance rates we report from WA and NT are comparable to those previously reported^26^.

Clindamycin (and related macrolide) resistance rates in MRSA are alarmingly high^27^. Twenty six percent of all MRSA isolates (35% in blood and 25% in swab isolates) in our NT analysis were resistant to inducible-clindamycin. This finding is a local impediment to empirical use of this agent for both community MRSA and as a synergistic antibiotic for severe skin and soft tissue infections in hospital patients. While inducible clindamycin rates were high, clindamycin may still have a broader efficacy than suggested, especially where macrolide use is limited and therefore macrolide-related induction of clindamycin resistance is potentially minimized. This is indeed the case for some regions, but the widespread use of azithromycin for therapy of particular infections (i.e. sexually transmissible infection) across the regions and for trachoma in selected locations^28^ cautions against routinely using clindamycin when inducible clindamycin resistance is reported. These data will be used to inform revisions of local treatment guidelines^28^. Higher clindamycin resistance rates still, at up to 44% among community MRSA isolates, have been reported in the southern half of the NT, a region of the jurisdiction that did not participate in this study^29^. Reassuringly however, a marginal decrease in inducible-clindamycin resistance was seen over time and a more evident decrease occurred across all jurisdictions during the study period. While this study was not designed to explain this trend, we speculate that, in contrast to key drivers of methicillin resistance, this may reflect decreasing prescribing rates of erythromycin in community clinics and guideline-restriction of azithromycin use.

Antibiotic resistance does not always inexorably increase over time but rather, fluctuates according to factors including changing prescribing habits and transmission dynamics^30,31^. We found that changes in the proportion of MRSA were regionally concentrated and did not follow a gradient across the geographical area included in the study. This focal nature particularly, in the west part of Northern Australia is suggestive of MRSA outbreaks with community transmission in confined niches and is less likely to suggest regional differences in prescribing practices driving AMR. This is supportive of the growing body of evidence that points to contagion (i.e., spread) being the major but frequently under-appreciated factor driving the increased prevalence of AMR^32^. That the NT has by far the highest rates of overcrowding per household in Australia^33^ may in part explain why the Northern Territory, despite using fewer prescriptions for some conditions^12^, continues to demonstrate high rates of AMR as we report in this study. Unpacking the social factors contributing to transmission and spread of AMR is important both for clinical management of patients and to mitigate risks of further spread.

Limitations of this study include that not all regional pathology providers participated, and clinical information for each isolate was unavailable. However, the data presented are more comprehensive, and inclusive of primary care data, than any previous reports for this region that are published. Sampling bias may have favoured sample collection (especially swabs) from treatment failure cases, hence may have over-estimated the proportion with AMR. Smaller number of blood isolates in NT and WA may also incorrectly inflate AMR proportions in those regions. Indeed, local treatment guidelines for remote settings with limited laboratory access recommend swab testing of skin infections only if initial empirical therapy fails^28^. Data have only been entered up to 2017 and further updates will follow in late 2020. The existing information provides regionally-specific figures from which current rates can be approximated. Lastly, comparison of data from participating laboratories should be approached with some caution due to the use of either CLSI or EUCAST methods over time. However, both methods are internationally recognised, and any differences are well documented.

We provide evidence using *S. aureus* as an example of a model of AMR disease surveillance delivered using a digital platform, that is both feasible and operative. Our study describes geospatial epidemiology of infection with *S. aureus* in a region that is under-served, suffers from a high disease burden and is geographically isolated. Successful implementation of this platform and deployment into clinical practice planned for in 2020 is turning data-rich systems into information-rich ones and innovating the way health services receive and act on changing patterns of resistance. The HOTspots platform is currently being evaluated, updated to include more sophisticated high-quality reports and is planned to have ongoing 6-monthly data updates from late 2020. The challenge will be to sustain efforts already initiated and to percolate to next levels of government, underpinning the critical need for coordinated AMR surveillance and response in Australia. This approach is scalable and transferrable to other settings and other disease surveillance programs. Given the significant health burden of AMR and infectious disease which continues to increase in this region and neighbouring low and middle-income countries, the benefits of early detection, timely and appropriate response hold great promise to expand this platform into other settings.
